# How to manage malignant tumors surgery in patients supported *with* long term implantable left ventricular assist device?

**DOI:** 10.1186/1749-8090-8-S1-O153

**Published:** 2013-09-11

**Authors:** H Smail, JM Baste, K Guernon, C Fister, JP Bessou, PY Litzler

**Affiliations:** 1 Department of Thoracic and Cardiovascular Surgery, Rouen University Hospital Charles Nicolle, Rouen, France; 2 Department of Anesthesiology, Rouen University Hospital Charles Nicolle, Rouen, France; 3 Department of Urology, Andrology and Renal Transplantation, Rouen University Hospital Charles Nicolle, Rouen, France

## Background

Left ventricular assist devices are increasingly used as bridges to transplantation. Malignant tumors can be discovered on the pre-implantation or pre-transplantation screening and surgeons may be required to perform tumors resection in those patients. We reported our experience on this challenge given the hemodynamic, hematologic and outcome considerations.

## Methods

34 patients underwent implantation of Heartmate II (HMII); we performed 4 resections of malignant tumors: 2 partial nephrectomies for renal tumors (8 months after implantation), a left segmentectomy for lung cancer (2 years later) under videothoracoscopy and a tumorectomy with lymphadenectomy for breast cancer (9 months later). Renal tumors were diagnosed during the pre-implantation screening; while the others were diagnosed after the device’s implantation.

## Results

Invasive surgical approach can be difficult in patients with heart failure; therefore we decided to support them with HMII before the renal tumor’s resection. Surgery in patients supported with HMII device expose to bleeding as well as hemostasis revealed an acquired Von Willebrand disease in 2 patients. 5 days prior to the surgery, we substituted Vitamin K antagonist with heparin (PTT1,5- 2). None of them experienced bleeding. During surgery, the use of the right lateral decubitus in 3 patients resulted in decreased cardiac output, which was resolved with vasopressor, hydration and decrease of the pump speed. Length of stay in intensive care unit was 1-4 days and reoperation was necessary for evacuation of post nephrectomy hematoma. After discussion with cardiologists and oncologists, 1 patient received heart transplant 2 years after partial nephrectomy and complete remission;3 are on the waiting list.

## Conclusion

Our results suggest that, in the setting of cancer in a pretransplant candidate, tumors resection can be safely achieved even for high-risk HMII patients and regaining candidacy for heart transplant is conceivable.

